# Automated Laser-Fiber Coupling with Three-Axis Fiber-Core Alignment for Optical-Resolution Photoacoustic Microscopy

**DOI:** 10.3390/s26206469

**Published:** 2026-10-13

**Authors:** Yongjae Jeong, Dongbin Lee, Haeni Lee, Hwidon Lee, Jinwoo Yoo, Jeesu Kim

**Affiliations:** 1 Department of Cogno-Mechatronics Engineering, School of Transdisciplinary Engineering, Pusan National University, Busan 46421, Republic of Korea; zxc2726655@pusan.ac.kr (Y.J.); ehdqls3367@pusan.ac.kr (D.L.); haenilee@pusan.ac.kr (H.L.); hwidonlee@pusan.ac.kr (H.L.); 2 Engineering Research Center for Color-Modulated Extra-Sensory Perception Technology, Pusan National University, Busan 46241, Republic of Korea; 3 Department of Automobile and IT Convergence, Kookmin University, Seoul 02707, Republic of Korea; jwyoo@kookmin.ac.kr

**Keywords:** fiber coupling, photoacoustic microscopy, automatic, optical fluence

## Abstract

Optical-resolution photoacoustic microscopy has become an important biomedical imaging modality for visualizing optical absorption contrast with micrometer-scale spatial resolution in superficial tissues. Because this modality relies on tightly focused laser illumination, precise optical alignment is essential, and free-space-to-fiber laser coupling is one of the most critical alignment steps. This issue becomes particularly important in transportable optical-resolution photoacoustic microscopy systems, where repetitive manual readjustment is required. Although our previous automated coupling approach based on motorized mirror control reduced the need for manual beam-path alignment, its optimization capability was limited when the focal position relative to the fiber core is not actively controlled. In this study, we propose an upgraded automated fiber-coupling module that integrates beam steering and fiber-side focusing alignment. In addition to the incident beam path control using motorized mirrors, a piezo-driven three-axis stage carries the coupling objective lens to align the focused beam with the fiber core. The coupled light was divided into imaging and monitoring paths, enabling real-time feedback-based optimization. The proposed module was validated by achieving high-quality photoacoustic images of both phantom and biological tissue through automated coupling. By extending automated coupling from mirror-based beam steering alone to combined beam steering and three-axis fiber-core alignment, the proposed coupling module provides a practical solution for improving the robustness, usability, and transportability of optical-resolution photoacoustic microscopy platforms and other fiber-coupled optical imaging systems.

## 1. Introduction

Photoacoustic (PA) imaging is a hybrid biomedical imaging modality that combines optical absorption contrast with ultrasound detection, enabling functional and molecular imaging of biological tissues with scalable spatial resolution and imaging depth [1,2,3]. In PA imaging, short pulsed lasers are delivered into biological tissue, and the thermoelastic expansion of light-absorbing chromophores generates acoustic waves that are detected to form an image [4,5]. Depending on the imaging scale and purpose, PA imaging can be implemented as PA tomography (PAT) for macro-scale small-animal or human imaging [6,7,8,9,10], or PA microscopy (PAM) for high-resolution micro-scale imaging [11,12,13]. Among PAM configurations, optical-resolution PAM (OR-PAM) achieves micrometer-scale lateral resolution by using tightly focused optical illumination, allowing label-free visualization of microvascular structures such as individual capillaries in superficial tissues [14,15,16,17]. Owing to these advantages, OR-PAM has been widely applied in biomedical studies, including neuroscience, dermatology, ophthalmology, and oncology [18,19,20]. Because the PA signal amplitude is proportional to the optical fluence delivered to the sample, the efficiency and stability of light delivery directly affect the signal-to-noise ratio (SNR) and resulting image quality.

Fiber-based light delivery has become an increasingly attractive strategy for OR-PAM, as it separates the bulky excitation laser from the scanning head and provides a flexible and reconfigurable optical path [21,22]. This flexibility has enabled various compact and mobile OR-PAM implementations, ranging from handheld probes [23,24] to miniaturized systems for freely moving animals [25,26]. More recently, this trend has further extended to transportable cart-based platforms designed to operate outside the constraints of a fixed optical table [27]. In parallel, advanced scanning modules have expanded OR-PAM capabilities toward high-speed, wide-field, and functional hemodynamic imaging [28,29]. Collectively, these developments reflect a broader effort to transform OR-PAM from a laboratory-bound imaging instrument into a more practical and deployable tool for preclinical and clinical applications. However, this increasing reliance on fiber-based light delivery also makes the stability of free-space-to-fiber coupling a critical factor for maintaining consistent imaging performance [30,31].

Efficient coupling into a single-mode fiber (SMF) requires precise alignment of the focused excitation beam to the micrometer-scale fiber core in both the lateral and axial directions. Because of this stringent requirement, small misalignments can substantially reduce the coupled optical power and the delivered fluence [32,33,34,35]. In particular, in mobile OR-PAM systems, such alignment can be easily disturbed by system relocation, mechanical vibration, and thermal drift. Once the alignment is perturbed, recovery of the optimal coupling condition generally depends on manual, iterative readjustment, which is time-consuming, operator-dependent, and impractical for routine operation. Therefore, a method that can automatically restore the optimal coupling condition without operator intervention is required for robust and practical mobile OR-PAM systems.

Automated fiber alignment has been extensively investigated in other optical applications. In photonic packaging, multi-axis motorized or piezoelectric stages combined with optical-power feedback have been used for active alignment between optical fibers and photonic integrated circuits or waveguides [36,37,38]. Similarly, adaptive fiber-coupling systems for free-space optical communication have employed piezoelectric fiber positioning, beam steering, and optimization algorithms [39,40,41]. Commercial alignment platforms also provide multi-axis positioning for automated fiber or waveguide alignment [42]. However, these systems are primarily designed for photonic assembly, device testing, or dynamic free-space communication links and rely on application-specific control architectures. Their direct integration into OR-PAM systems, where the laser beam must be reliably coupled into a single-mode fiber while maintaining a compact and transportable imaging configuration, remains limited.

To address this limitation, we previously demonstrated an automated coupling approach based on motorized mirror control, which optimized fiber coupling by steering the incident beam path without manual intervention [43]. Although this method effectively reduced the need for manual beam-path alignment, it controlled only the direction and lateral position of the incident beam and did not actively regulate the focal position relative to the fiber core. As a result, its optimization capability was limited when the focal spot shifted axially with respect to the fiber core. This limitation is consistent with Gaussian mode coupling theory, in which coupling efficiency *T_δ_* decreases with axial offset (*δ*) between the focused beam waist and the fiber mode as follows [44]:

(1) $$T_{\delta }\approx \exp\left(-\frac{4\delta^{2}}{k^{2}{\left(\omega_{i}^{2}+\omega_{f}^{2}\right)}^{2}}\right),$$

where *ω_i_* and *ω_f_* are the beam-waist radii of the incident beam and the fiber mode, respectively, and *k* = 2*π*/*λ* is the optical wavenumber, with *λ* denoting the optical wavelength. Because the axial defocus originates from a mismatch between the focal plane and the fiber facet, it cannot be fully compensated by adjusting the incident beam direction or lateral position alone. Therefore, achieving stable and optimal coupling requires active three-dimensional alignment of the focused beam relative to the fiber core.

In this study, we propose an upgraded automated fiber-coupling module that integrates beam steering with active fiber-side focusing alignment. A three-axis stage carrying the coupling objective lens aligns the focused beam with the fiber core in three dimensions, while two motorized mirrors control the incident beam path. A portion of the coupled light is continuously measured by a photodetector (PD) and used as real-time feedback for closed-loop optimization. The proposed module was validated through PA imaging of phantom and biological tissue, in which the increased coupled optical power achieved by automated alignment was reflected in improved PA amplitude and SNR. By extending automated coupling from mirror-based beam steering alone to combined beam steering and three-axis fiber-core alignment, the proposed module offers a practical method for improving the robustness, usability, and transportability of OR-PAM platforms and other fiber-coupled optical imaging systems.

## 2. Materials and Methods

### 2.1. System Configuration

#### 2.1.1. Automated Laser-Fiber Coupling Module

**Figure 1:**
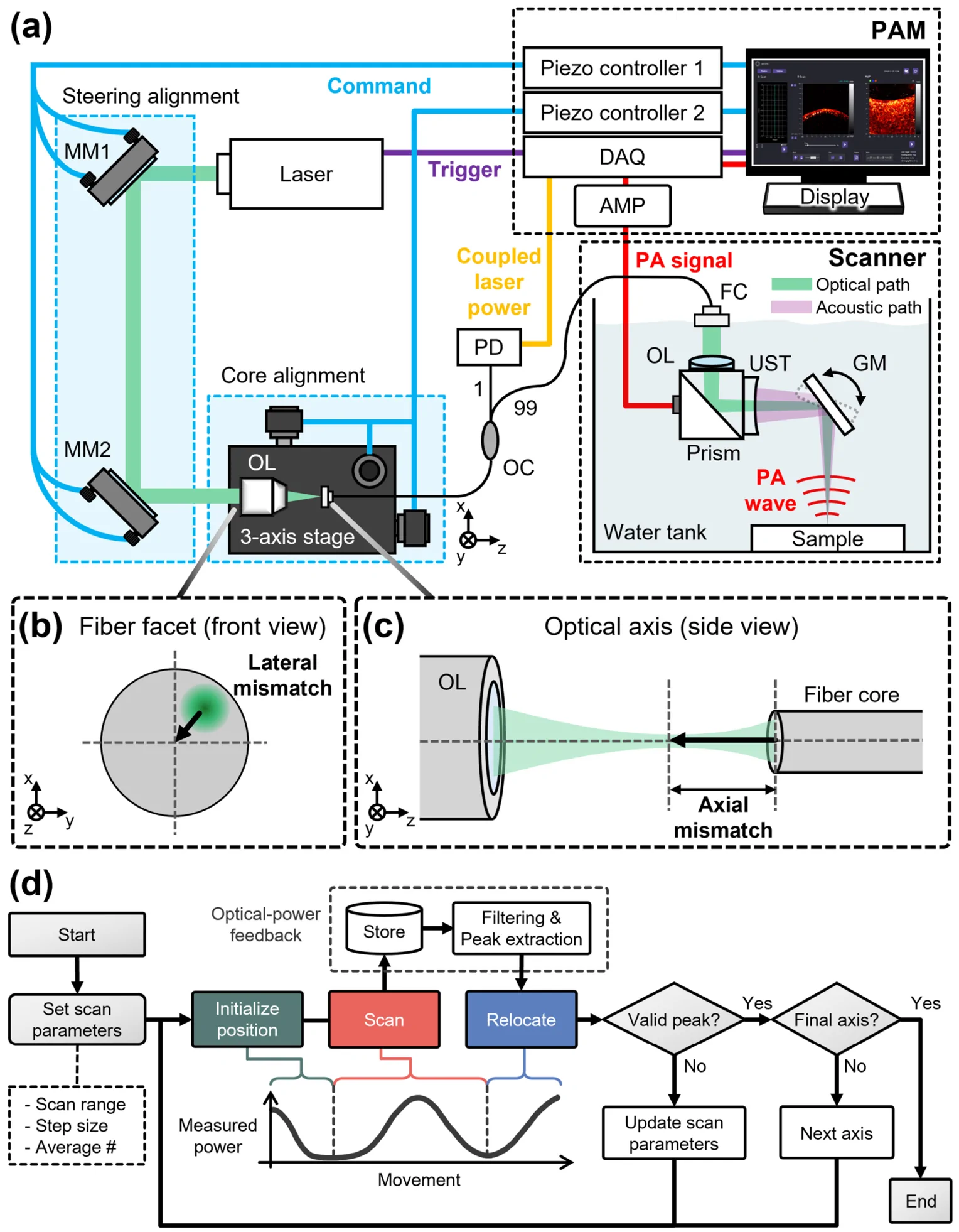
Schematic illustration of the automated laser-fiber coupling system integrated with an OR-PAM system. (**a**) Overall system configuration. Two MMs steer the incident laser beam, while a piezo-driven three-axis stage carrying the coupling OL adjusts the focal position relative to the fiber core. A small fraction of the coupled light is directed to a photodetector for real-time optical-power feedback, while the remaining light is delivered to the OR-PAM imaging module. The measured optical power is used for automated coupling optimization, while the remaining light is delivered to the OR-PAM imaging module. (**b**) Lateral mismatch between the focused beam spot and the fiber core at the fiber facet. (**c**) Axial mismatch between the focal plane and the fiber facet along the optical axis. (**d**) Feedback-based control algorithm for sequential alignment, including scanning, peak detection, relocation, and transition to the next axis. PA, photoacoustic; OR-PAM, optical-resolution PA microscopy; MM, motorized mirror; OL, objective lens; OC, optical coupler; PD, photodetector; FC, fiber collimator; GM, galvanometer mirror; UST, ultrasound transducer; AMP, amplifier; DAQ, data acquisition module.

The automated laser-fiber coupling module consisted of a pulsed laser source, a beam steering alignment unit controlling the incident beam path, a core alignment unit aligning the focal spot of the beam to the fiber core, and a monitoring path for real-time measurement of the coupled optical power (Figure 1a, Table S1). A pulsed laser (DX-532-2, Photonics Industries, Ronkonkoma, NY, USA) emitting light with a wavelength of 532 nm, a pulse width of approximately 6 ns, and a pulse repetition rate of 20 kHz was used as the light source.

The beam steering alignment unit followed the previously reported setting [43], in which two motorized mirrors (PIM1, Thorlabs, Newton, NJ, USA) mounted optical mirrors (PF10-03-G01, Thorlabs, USA) and were driven by a piezo motor controller (KIM101, Thorlabs, USA). The horizontal and vertical angles of each mirror were independently controlled, constituting four control axes in total, with a minimum angular step size of 3 × 10^−5^ deg and an angular travel range of ±2 deg.

The core alignment unit consisted of an objective lens (LMH-20X-532, Thorlabs, USA) with a focal length of 10 mm and a numerical aperture (NA) of 0.4, mounted on a three-axis stage (MAX301, Thorlabs, USA). Each axis (*x*, *y*, and *z*) was driven by a piezo controller (KPZ101, Thorlabs, USA), providing a travel range of 20 μm and a minimum step size of 20 nm. In addition to the mirrors, which controlled the direction and position of the incident beam, the three-axis stage translated the objective lens to adjust the focal spot relative to the fiber core.

This configuration compensated for two types of alignment error. First, the lateral misalignment between the focal spot and the center of the fiber core was corrected through coarse beam steering using the mirrors and fine x–y translation of the objective lens (Figure 1b). Second, the axial misalignment between the focal plane and the fiber facet was corrected by z-axis translation of the stage (Figure 1c). Consequently, the system provided seven control axes for fiber coupling: four mirror-based steering axes and three objective lens alignment axes.

The light focused by the objective lens was coupled directly into the input port of a 99:1 fiber optic coupler (TN532R1F1, Thorlabs, USA), which was based on SMF (460HP, Thorlabs, USA) with a mode field diameter of 3.5 μm and an NA of 0.13. Also, 1% of the coupled light was directed to a PD (S140C, Thorlabs, USA) and measured as the coupled optical power, while the remaining 99% was delivered to the OR-PAM module for image acquisition. This configuration enabled continuous monitoring of the coupling efficiency, which was subsequently used as a closed-loop feedback for alignment.

#### 2.1.2. Optical-Resolution Photoacoustic Microscopy

The 99% portion of the optical coupler was connected to a fiber collimator (F220FC-532, Thorlabs, USA) to deliver the coupled laser light to the imaging head of a commercial OR-PAM module (OptichoM, Opticho, Pohang, Republic of Korea). The light was then focused by an imaging objective lens and delivered to the sample through the central aperture of a custom-made ring-shaped focused single-element ultrasound transducer. By using this configuration, the optical and acoustic axes were coaxially aligned. A water-immersible scanning mirror was used to scan both optical and acoustic paths across the imaging area. All the sample and scanning components were immersed in a water tank for acoustic coupling.

The generated PA signal was detected by the ultrasound transducer, whose outer diameter and focal length were 7 mm and 18.6 mm, respectively. These characteristics correspond to an F-number of 2.66 at a center frequency of 25 MHz. The detected PA signals were amplified by a 20 dB amplifier and digitized for image reconstruction. The lateral resolution of the OR-PAM module used in this study has been reported to be approximately 10 μm [45].

### 2.2. Control and Signal Acquisition Sequences

All the hardware required for the automated coupling and the image acquisition was controlled by custom-developed LabVIEW-based software (LabVIEW 2021, National Instruments, Austin, TX, USA) running on a PC. The beam steering alignment unit and core alignment unit were driven through two piezo controllers, which were connected to the PC via a USB serial connection to receive the alignment commands.

For real-time monitoring of the coupling efficiency, the output of the PD connected to the 1% port of the OC was transferred to the computer through a power meter console (PM100D, Thorlabs, USA). The averaging time of the power meter was set to 0.5 ms, corresponding to approximately 10 laser pulses at the pulse repetition rate of 20 kHz. The specified measurement range of the PD is 1 μW to 500 mW with a display resolution of 1 nW, and values below this range were therefore used as relative indicators for the feedback-based alignment rather than as calibrated absolute powers.

During image acquisition, the PA signals were amplified and transferred to a data acquisition board (DAQ, ATS9352, AlazarTech, Pointe-Claire, QC, Canada), where they were digitized with a resolution of 12 bits at a sampling rate of 500 MS/s. The laser emission, the signal acquisition, and the scanning were synchronized by a trigger signal generated from the DAQ board. The acquired PA signals were transferred to the PC for image reconstruction.

### 2.3. Coupling Optimization Algorithm

The optimization of the coupling efficiency was performed by sequentially driving each control axis using the PD signal as feedback (Figure 1d). Before starting the optimization, the user entered the scan range and the step size. The *x* and *y* axes of the core alignment were scanned over a range of 30 V in 0.1 V increments, corresponding to 300 steps, a scan range of approximately 8 μm, and a step size of approximately 27 nm. Because the coupling efficiency varies more slowly along the axial direction than the lateral directions, the scan range of the *z*-axis was extended to 60 V (approximately 16 μm) while the step size was kept identical. Each mirror axis of the steering alignment was scanned over 800 steps at a rate of 200 steps/s, corresponding to a scan time of approximately 4 s per axis.

The optimization of each axis proceeded in the order of position initialization, scanning, and relocation (Algorithm S1). The initial position was set differently according to the driving characteristics of the two alignment units. Because the mirror axes of the beam steering alignment unit are driven by piezo motors and can move bidirectionally, each axis was initially moved to the negative direction by half of the scan range and was then scanned over the entire scan range in the positive direction. In contrast, the piezo actuators of the three-axis stage in the core alignment unit are driven by a unipolar voltage between 0 and 75 V, which defines one end of the 20 μm travel range. Accordingly, before the alignment, each axis was manually displaced in the negative direction by half of the scan range using the differential micrometers built into the stage, so that the initial alignment condition was located near the center of the subsequent scan.

During the scanning, the corresponding axis was moved by the step size while the measured PD signal at each position was stored. Once the scan was completed, a smoothing filter was applied to the stored signal to suppress the fluctuating noise, and the peak value of the signal was extracted. The axis was then driven to relocate to the condition corresponding to the extracted peak power. A valid peak was defined as a local maximum in the filtered optical-power profile, identified by a positive-to-negative zero crossing of the first derivative. If a valid peak was detected within the scan range, the optimization of the corresponding axis was considered complete, and the algorithm proceeded to the next axis. If no valid peak was detected, the scan parameters were updated, and the scan was repeated. The overall alignment sequence was terminated after successful optimization of the final axis.

### 2.4. Photoacoustic Imaging

#### 2.4.1. Phantom Preparation

A leaf skeleton was used to evaluate the laser-fiber coupling module. To minimize deformation, the leaf skeleton was fixed on a custom-designed mold and subsequently immersed in water for a sufficient time. Once the shape had been confirmed to be stable, the phantom was positioned at the focal plane of the objective lens by adjusting its axial position. PA images were then acquired over a 3 × 4 mm^2^ region with a step size of 10 μm along both the *x* and *y* axes.

#### 2.4.2. In Vivo Human Imaging

The microvascular network in a finger of a healthy volunteer was imaged. All human experiments were conducted according to a protocol approved by the Institutional Review Board of Pusan National University (PNU IRB/2023_144_HR). Written informed consent was obtained from the volunteer prior to the experiments. The maximum optical fluence delivered to the skin surface was 16.3 mJ/cm^2^, which is below the ANSI safety limit of 20 mJ/cm^2^ at 532 nm. To prevent eye damage, both the operator and the volunteer wore safety glasses (LG9, Thorlabs, USA) throughout the experiments. PA images were acquired over a 4 × 6 mm^2^ region with a step size of 10 μm along both the *x* and *y* axes.

#### 2.4.3. Signal and Image Processing

The acquired three-dimensional volume data were post-processed using custom software in MATLAB (R2024a, MathWorks, Natick, MA, USA). A band-pass filter (2.5–50 MHz) was applied to suppress noise outside the detection bandwidth of the ultrasound transducer. The envelope of the filtered signals was then extracted using the Hilbert transform, from which maximum amplitude projection (MAP) images were generated. Finally, a 3 × 3 median filter was applied to the MAP images to remove locally spurious noise.

For the in vivo images, an additional procedure was applied to remove strong PA signals at the skin surface. For each A-line, the depth index of the skin surface was determined by detecting the signal that exceeds a predefined threshold first. A median filter was additionally applied to remove abruptly deviating values. A predefined depth range below the detected skin surface was then assigned as the vascular region, and the maximum amplitude in this region was used to form MAP images. Identical processing parameters were applied to all images, indicating that the observed differences in signal amplitude and SNR are predominantly attributable to the optical power delivered through the fiber.

## 3. Results

### 3.1. Automated Laser-Fiber Coupling Optimization

To validate the ability of the proposed system to automatically recover laser-fiber coupling, the optimization was initiated from a decoupled condition in which the coupled optical power was substantially reduced. The decoupled condition was prepared by displacing the three-axis stage from the pre-aligned position, so that the focal spot was shifted away from the fiber core. The automated alignment was then performed sequentially along the three core-alignment axes followed by the four beam-steering axes. This order was adopted because the objective lens stage directly controls the three-dimensional position of the focused beam relative to the fiber core, whereas the motorized mirrors primarily adjust the incident beam path and angle. When the focal spot is substantially far from the fiber core, the optical-power feedback during mirror scanning could be ambiguous, making it difficult to identify the global optimum. Therefore, the beam was first brought close to the fiber core through three-dimensional core alignment, and the incident beam path was subsequently fine-tuned by mirror-based steering alignment.

**Figure 2:**
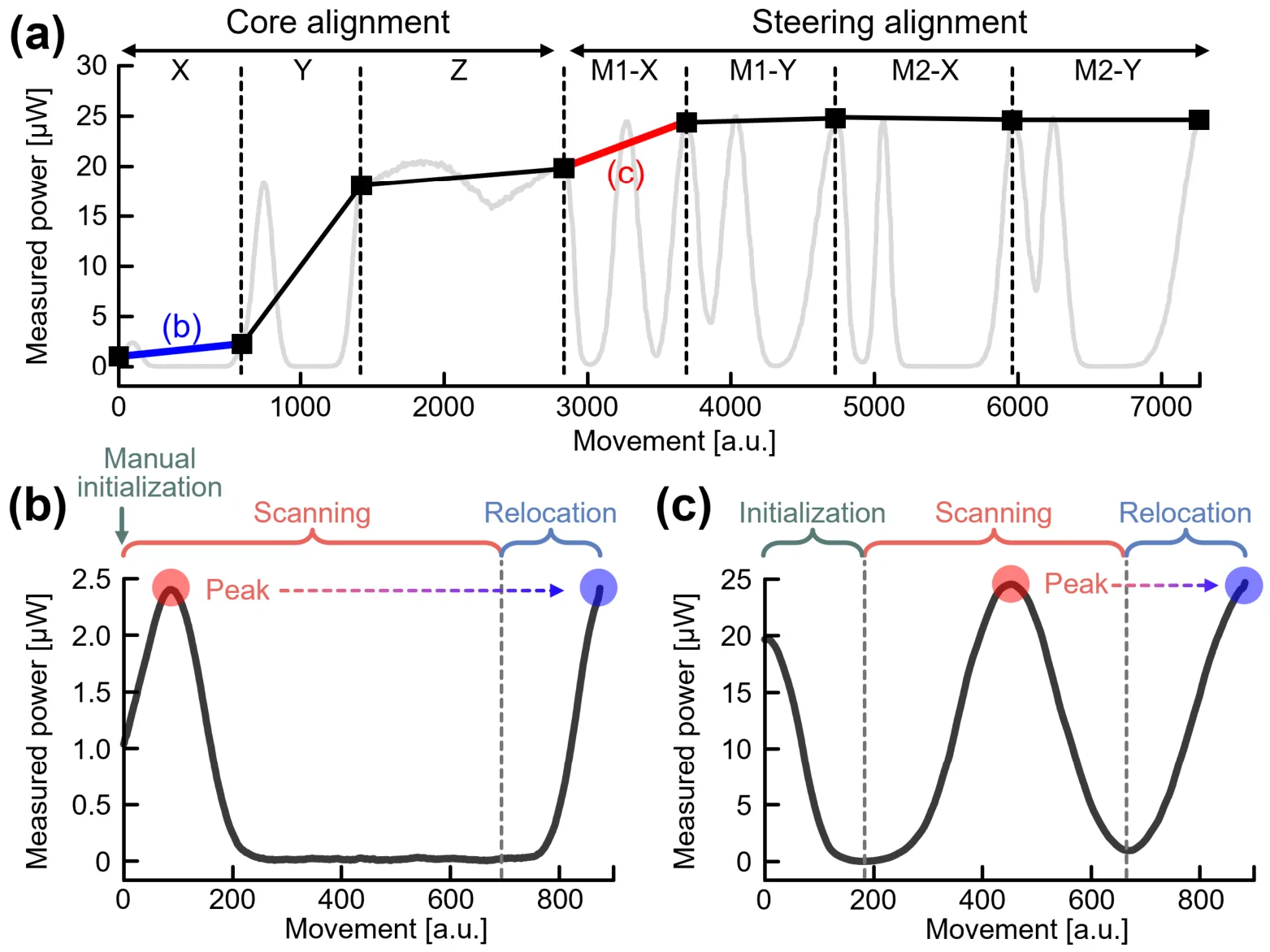
The measured optical power during automated laser-fiber coupling optimization. (**a**) Coupled optical power measured over the entire seven-axis alignment sequence. The alignment was performed sequentially along the three core-alignment axes of the objective-lens stage (X, Y, and Z), followed by the four steering-alignment axes of the motorized mirrors (M1-X, M1-Y, M2-X, and M2-Y). Dashed vertical lines indicate the boundaries between individual axis-alignment steps. The gray curve represents the raw optical-power feedback measured during scanning, and the black line represents the optimized optical power after relocation to the peak position for each axis. (**b**) Enlarged view of the first core-alignment step marked blue in (**a**), showing scanning and relocation to the peak position. (**c**) Enlarged view of the first steering-alignment step marked red in (**a**), showing initialization, scanning, and relocation to the peak position. M, mirror; X, *x*-axis; Y, *y*-axis; Z, *z*-axis.

The coupled optical power increased during the automated alignment (Figure 2a). Starting from approximately 1.2 μW at the initial position, the optical power increased to 2.4 μW after *x*-axis alignment, 17.9 μW after the *y*-axis alignment, and 19.7 μW after the *z*-axis alignment. During the subsequent steering alignment, 24.3 μW and 24.9 μW were measured after the *x*- and *y*-axes in the first mirror, respectively. In contrast, the *x*- and *y*-axes in the second mirror yielded 24.4 μW and 24.8 μW, respectively, with power variations remaining within 1 μW. Under this alignment sequence, a substantial increase in coupled optical power occurred during the core-alignment steps, followed by an additional increase during first-mirror steering. The second mirror produced only minor changes in the coupled optical power, indicating that the coupling condition was already close to the optimum by this stage. The entire seven-axis alignment sequence was completed in approximately 59 s, excluding the manual coarse initialization performed before core alignment. The coupling efficiency after the alignment was approximately 39%, consistent with the typical range reported for single-mode fiber delivery in previous PAM systems [46,47,48].

Representative axis-alignment profiles further confirmed that the feedback-based optimization successfully detected the peak coupling position during both core alignment (Figure 2b) and steering alignment (Figure 2c). In both cases, the measured optical power exhibited a distinct peak within the scan region, and the corresponding axis was relocated to the extracted peak position. For the core-alignment axes, the scan was preceded by a manual initialization step, in which the three-axis stage was shifted toward the negative direction because automatic negative-direction control was not available due to hardware limitations. In contrast, the steering-alignment axes were initialized automatically by the motorized mirror controller before scanning. After relocation, the measured optical power agreed with the maximum value observed during scanning, indicating that the PD-based feedback successfully restored the peak coupling position for each alignment axis.

Additional experiments were performed to further evaluate the effects of axis order and repeated alignment. Reversing the order of core and steering alignment changed the relative power improvement contributed by each alignment group. Although the alignment performed first generally produced the larger initial recovery, the final coupled optical powers remained comparable for the two tested sequences (Figure S1). This result also suggests that the core- and steering-alignment degrees of freedom are coupled rather than acting independently. In addition, repeating the core-alignment procedure after completion of the full seven-axis sequence did not produce a further increase in coupled optical power, supporting the conclusion that the initial single-pass sequence had already reached an optimal coupling condition within the tested scan range (Figure S2).

### 3.2. Automated Coupling from a Low Initial-Power Condition

To evaluate the capability of the automated alignment to recover coupling efficiency from a nearly decoupled state, the optimization sequence was performed under a low-power initial condition in which the coupled optical power was substantially reduced to 0.67 nW. This value is below the display resolution of the PD readout. The condition represents a practical situation in which the optical alignment is severely perturbed.

**Figure 3:**
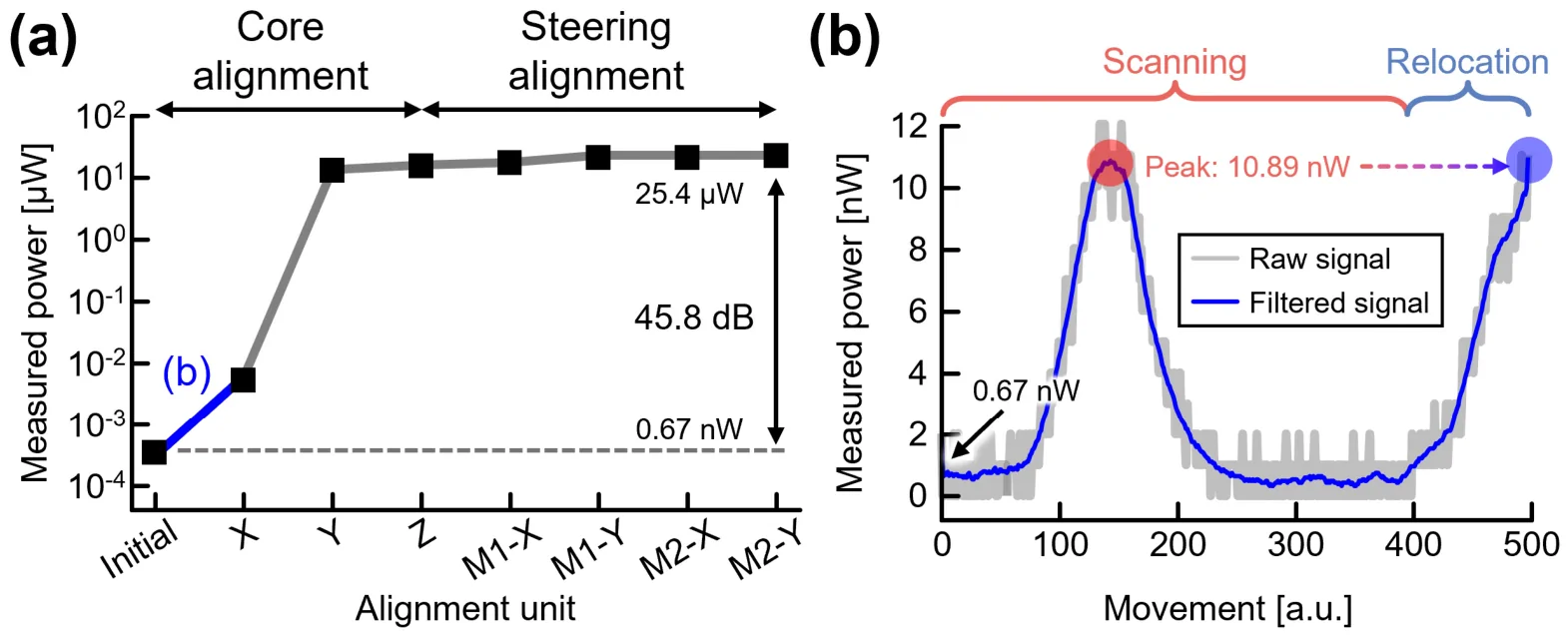
Recovery of coupling efficiency from a nearly decoupled initial condition using the automated alignment system. (**a**) Coupled optical power measured during the automated alignment sequence. The initial coupled optical power was 0.67 nW, and the power increased to 25.36 µW, corresponding to an improvement of 45.8 dB. The dashed horizontal line indicates the initial power level. (**b**) Raw and filtered optical power during the first core-alignment axis marked in blue in (**a**). The raw signal appeared as discrete because of the limited PD readout resolution, whereas the filtered signal revealed a single-peak profile. M, mirror; X, *x*-axis; Y, *y*-axis; Z, *z*-axis.

The automated alignment successfully restored the coupled optical power from the nearly decoupled state (Figure 3a). Starting from 0.67 nW, the coupled optical power increased to 18.1 μW after the core alignment and further increased to 25.4 μW after the steering alignment. The total improvement was 45.8 dB, corresponding to more than four orders of magnitude of recovery. In this experiment, substantial recovery occurred during the core-alignment steps performed first, followed by further improvement during steering alignment. These results demonstrate that the combined seven-axis alignment can recover coupling from a nearly decoupled condition.

The profile of the first core-alignment axis demonstrated how the feedback-based algorithm identified the coupling peak (Figure 3b). Owing to the limited display resolution of the PD, the raw optical power appeared as discrete step-like values. Therefore, the underlying continuous power profile was estimated by applying a smoothing filter, and the peak position was extracted. Relocation to this peak position increased the coupled optical power from the initial 0.67 nW to 10.89 nW. These results demonstrate that the proposed system can recover laser-fiber coupling from a nearly decoupled condition when a resolvable peak profile is present within the scan range. However, this value should not be interpreted as the minimum recoverable optical power of the system, because systematically preparing and controlling lower initial coupling conditions is experimentally difficult. Instead, the results confirm that the proposed automated laser-fiber coupling module is feasible over more than four orders of magnitude of coupled power without additional manual pre-alignment beyond the coarse offset applied before the alignment.

### 3.3. Phantom Validation: Photoacoustic Imaging of a Leaf Skeleton

**Figure 4:**
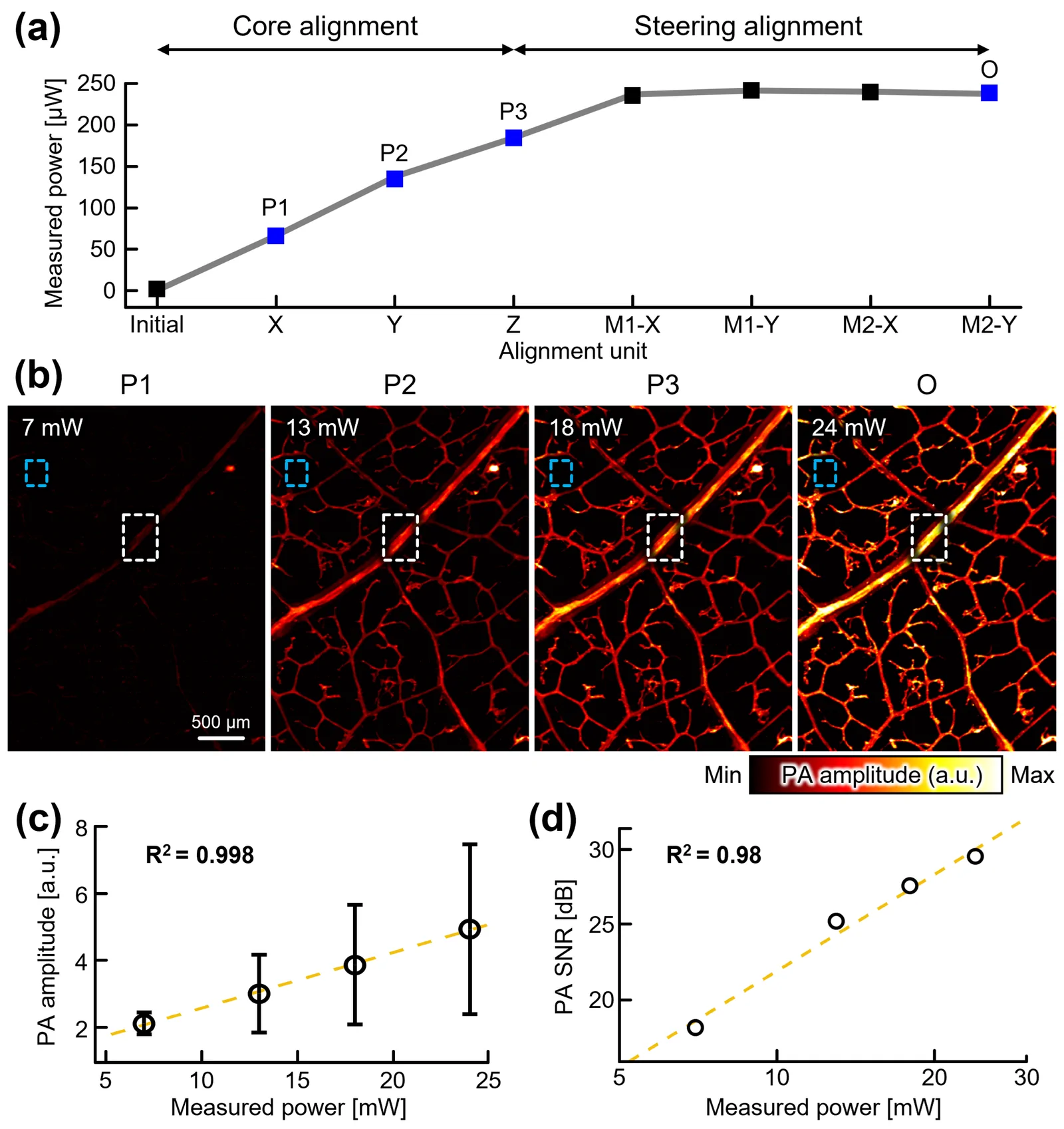
Phantom validation of the automated laser-fiber coupling module. (**a**) Coupled optical power measured during the automated alignment sequence. Blue markers indicate the four image acquisition points. (**b**) PA images of a leaf-skeleton phantom acquired at each imaging point. The white and blue dashed boxes indicate the signal and background regions of interest, respectively. (**c**) The average and standard deviation of PA amplitude in the signal region as a function of delivered optical power. (**d**) SNR of PA images according to delivered optical power. The yellow dashed lines are the first-order polynomial regression fits. PA, photoacoustic; SNR, signal-to-noise ratio. M, mirror; X, *x*-axis; Y, *y*-axis; Z, *z*-axis.

To verify that the increase in coupled optical power translates into improvement in PA image quality, PA images of a leaf-skeleton phantom were acquired at four selected points during the automated alignment sequence (Figure 4a). The leaf skeleton was used because its branching network is structurally similar to a vascular network. It also provides sufficient optical absorption at 532 nm and remains stationary during repeated imaging in the same region [49,50]. Because no detectable PA signal was observed at the initial decoupled state, the imaging points were selected as the peak points after each core alignment and the optimal point after the entire sequence. The optical powers measured by the PD at these points were 67, 135, 185, and 238 μW, corresponding to pulse energies of approximately 0.35, 0.65, 0.90, and 1.20 μJ delivered to the sample and coupling efficiencies of 11.1%, 22.3%, 30.5%, and 39.3%, respectively.

The PA image quality improved progressively as the delivered optical power increased (Figure 4b). At 7 mW, only the thick main vein was faintly visible, while the surrounding fine branches were difficult to distinguish from the background. At 13 mW, the reticulate venation structure became visible across the imaging area, although several distal branches remained fragmented. At 18 and 24 mW, the fine branches were resolved continuously, and the contrast between the venation and the background became increasingly distinct. As a result, the vascular-like hierarchy from the main vein to smaller branches was clearly visualized.

For the quantitative analysis, signal and background regions were defined as regions of interest (ROIs). Pixels within the signal ROI were considered PA signals only when their amplitude exceeded a threshold *τ_S_*, which was determined by the background ROI as follows:

*τ*_S_ = *μ*_B_ + 3 ∙ *σ_B_*, (2)

where *μ_B_* and *σ_B_* are the mean and the standard deviation of the PA amplitude within the background ROI. This threshold corresponds to a level exceeding 99.7% of the background noise distribution, assuming a Gaussian distribution. The mean PA amplitude of the pixels above the threshold, after subtraction of *μ_B_*, increased from 2.1 at 7 mW to 4.9 at 24 mW, corresponding to a 2.3-fold increase (Figure 4c). A first-order polynomial regression showed a strong linear relationship between the delivered optical power and the mean PA amplitude (R^2^ = 0.998). The standard deviation of the amplitude also increased with the delivered optical power, reflecting the larger amplitude variation within the signal ROI as both the main vein and weaker fine branches become detectable. Under the present experimental conditions, repeated laser exposure and water immersion did not produce an observable decrease in the PA response of the leaf-skeleton phantom during the measurement period.

The SNR of each image was calculated as the ratio between the background-subtracted mean PA amplitude of the signal pixels and the standard deviation of the background. The SNR increased from 18.4 dB to 29.9 dB, showing a linear dependence on the delivered optical power, with a slope of 21.4 dB/mW and R^2^ of 0.98 (Figure 4d). Throughout the four imaging points, *σ_B_* remained nearly constant, indicating that the SNR improvement mainly resulted from the increase in PA signal amplitude rather than a change in the background noise level.

These results confirm that the increase in coupled optical power achieved by the automated alignment was directly reflected in improved PA amplitude and SNR. Because both quantities increased linearly with the delivered optical power, the optical power measured by the PD can be a practical feedback variable for optimizing laser-fiber coupling.

### 3.4. Repeatability and Robustness of the Automated Alignment

**Figure 5:**
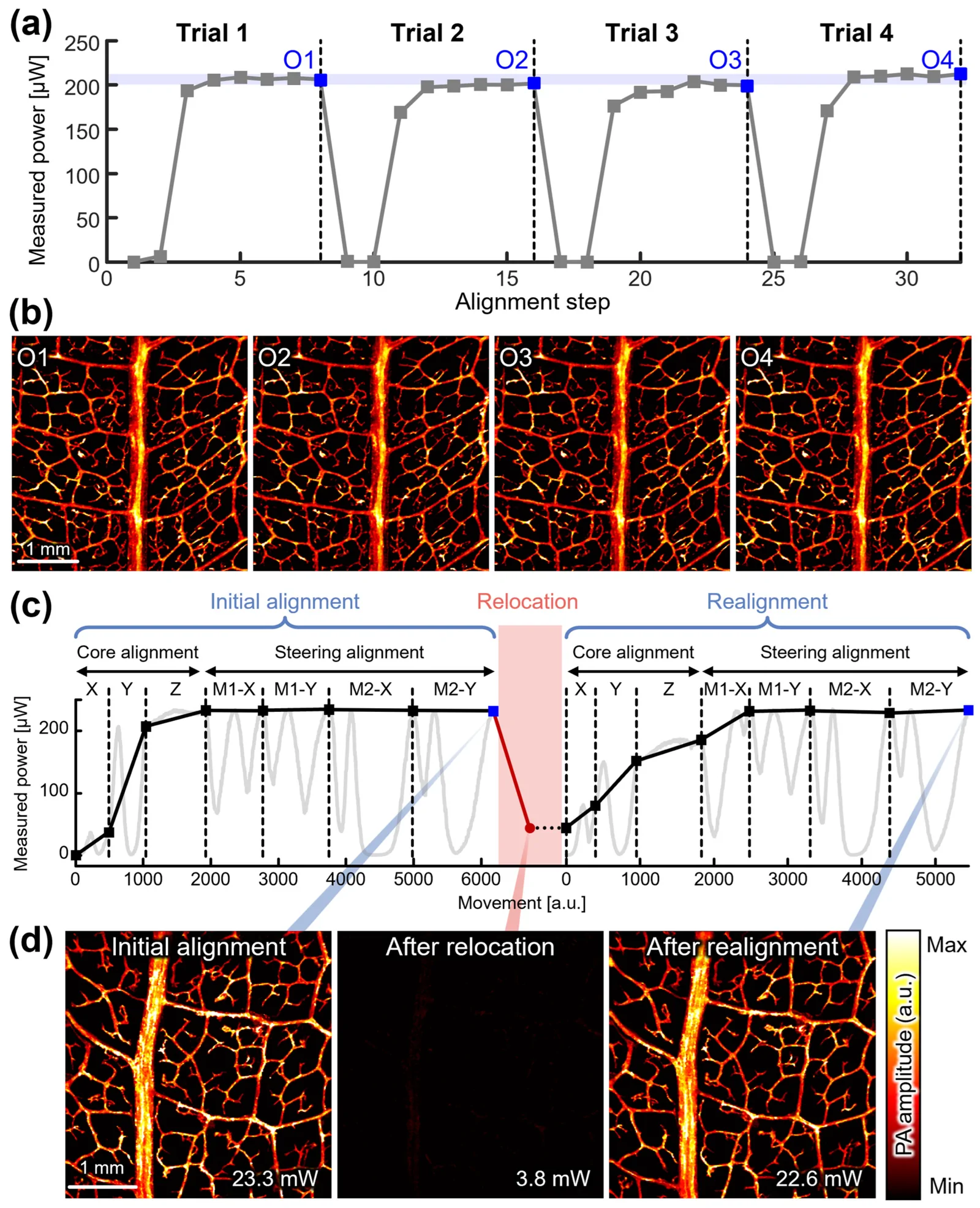
Repeatability and robustness of the automated laser-fiber coupling alignment. (**a**) Coupled optical power measured during four repeated alignment trials. Each trial was initiated from a different misaligned condition. Blue markers indicate the optimized points after each trial. The blue shaded area represents a 2.5% variation of the final coupled optical power, confirming repeatability of the automated alignment. (**b**) PA images of the leaf skeleton phantom acquired at the optimized point of each repeated trial. (**c**) Coupled optical power measured during initial alignment, relocation, and realignment. (**d**) PA images of the leaf skeleton phantom acquired after initial alignment, after relocation, and after automated realignment. PA, photoacoustic; O, optimized point.

To evaluate the repeatability and robustness of the automated alignment, the same alignment sequence was repeatedly performed after displacing the alignment axes (Figure 5a). The automated alignment was repeated four times under identical alignment parameters, including the scan range and the step size, while each trial started from a different initial misalignment condition. In all four trials, the coupled optical power was restored within a single seven-axis alignment sequence without requiring an additional pass. The optical powers measured at the optimized condition of each trial were 205.0, 201.3, 198.2, and 210.1 µW, corresponding to coupling efficiencies of 37.8%, 37.1%, 36.5%, and 38.7%, respectively. Although the extent and direction of the initial misalignment differed among trials, the final coupled power converged within a narrow range. The PA images acquired after each alignment also showed consistent vascular-like network structures and image contrast, confirming that the repeated automated alignment reproduced equivalent imaging performance (Figure 5b).

To further evaluate the robustness of the proposed module under practical transport conditions, an additional relocation experiment was performed using the previously reported transportable OR-PAM system (Figure S3) [27]. The optical coupling was monitored during initial alignment, relocation over a distance of approximately 50 m, and realignment (Figure 5c). At the initial location, the coupled optical power was 235 μW. After relocation without intentionally disturbing the individual alignment axes, the coupled optical power decreased to 38 μW, indicating substantial degradation of the coupling condition. The automated alignment procedure subsequently restored the coupled optical power to 228 μW, which was comparable to the value measured at initial alignment. The corresponding leaf-skeleton phantom images also showed recovery of the PA signal after realignment (Figure 5d), supporting the feasibility of restoring laser-fiber coupling after practical system relocation.

### 3.5. In Vivo Validation: Photoacoustic Imaging of a Human Finger

**Figure 6:**
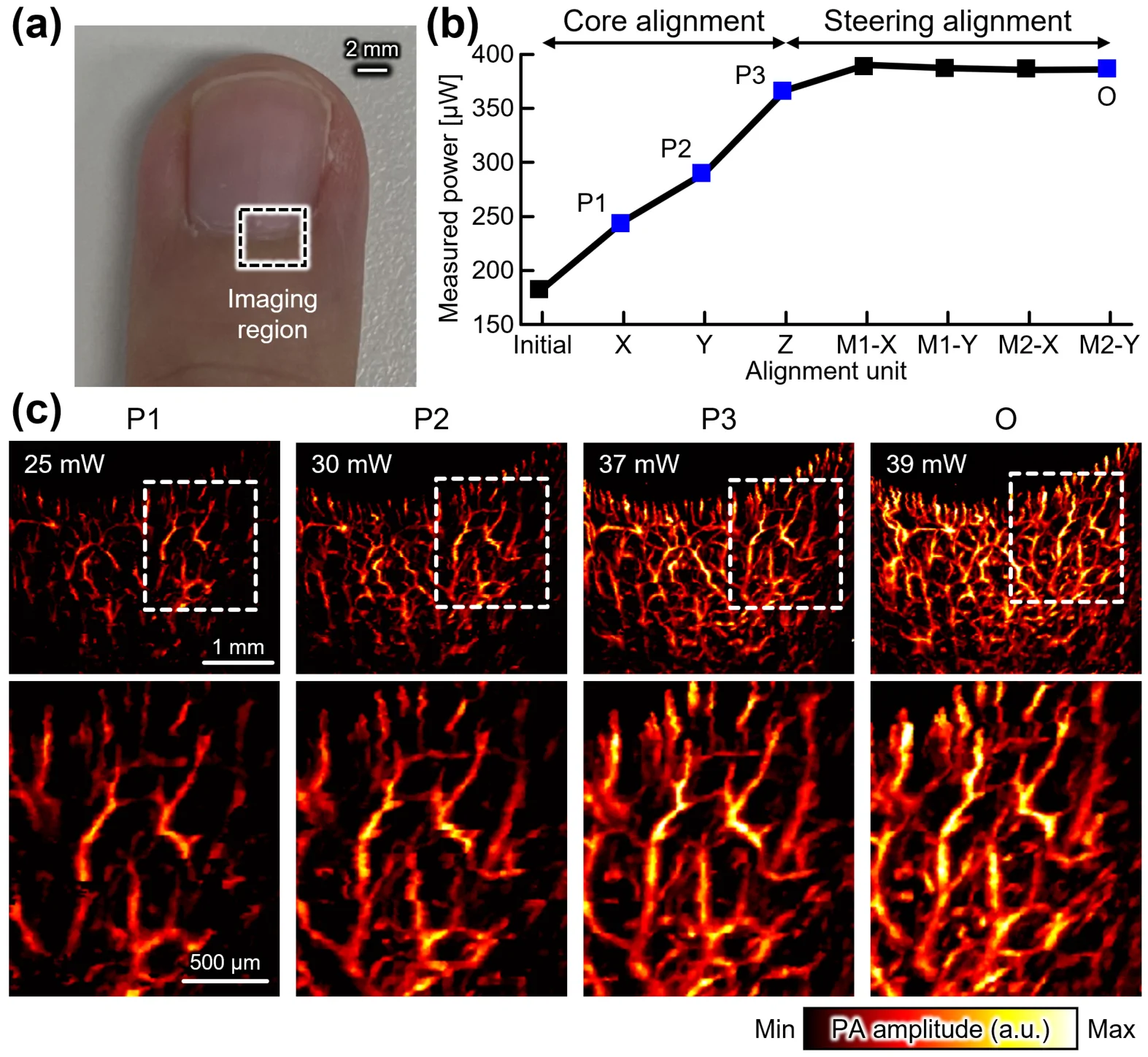
In vivo validation of the automated laser-fiber coupling module. (**a**) Photograph of the ring finger of a healthy volunteer, with the dashed box indicating the imaging region. (**b**) Coupled optical power measured during the automated alignment sequence. Blue markers indicate the four selected image acquisition points. (**c**) PA images of the human finger acquired at each imaging point. The white dashed boxes indicate the regions shown in the magnified images below. PA, photoacoustic; P, peak point; O, optimized point; M, mirror; X, *x*-axis; Y, *y*-axis; Z, *z*-axis.

To confirm the applicability of the proposed automated coupling system to in vivo imaging, PA images of the ring finger of a healthy volunteer were acquired at selected points during the automated alignment sequence, similar to the previous phantom validation (Figure 6a). The automated alignment was paused at four selected points corresponding to the completion of the *x*-, *y*-, and *z*-axes core-alignment steps and the completion of the entire sequence (Figure 6b). The laser powers delivered to the sample were approximately 25, 30, 37, and 39 mW, corresponding to pulse energies of approximately 1.25, 1.50, 1.85, and 1.95 μJ and coupling efficiencies of 24.9%, 29.8%, 36.8%, and 38.8%, respectively. During image acquisition, the finger was supported by a fixation mold to minimize motion-related artifacts.

The PA image quality improved as the delivered optical power increased (Figure 6c). At 25 mW, large superficial vessels were visible, whereas surrounding fine vessels exhibited relatively low contrast, making it difficult to trace their continuous courses. As the laser power increased, fine vascular structures became visible, and the vessel contrast gradually increased. At the highest delivered power of 39 mW, branching structures of the fine vessels and the deeper vascular features were more clearly visualized. The same tendency was observed in the magnified views, indicating that the relationship between the delivered optical power and PA image quality observed in the phantom experiment was also reproduced in biological tissue, where optical scattering and motion-related factors may be present. These results demonstrate that the proposed automated coupling system can improve the PA image quality not only in phantoms but also in biological tissue.

## 4. Discussion

In this study, we developed and validated an automated laser-fiber coupling module that integrates three-axis fiber-core alignment with mirror-based beam steering. The proposed design directly addresses a key limitation of fiber-coupled OR-PAM systems, in which small deviations in the focal position can substantially reduce the coupled optical power and degrade PA image quality. Compared with our previously reported automated-mirror-only system [43], which adjusted the incident beam direction and lateral beam position but could not fully compensate for axial mismatch between the focal plane and the fiber facet, the present system expands automated coupling to seven-axis alignment by adding three-dimensional control of the focal position relative to the fiber core (Table S2). Although the lateral core-alignment axes generally contributed more strongly than the axial axis because of the broader axial tolerance, the added z-axis provides an important capability for correcting focal-plane mismatch that cannot be directly compensated by mirror-based steering.

The optimization strategy was also updated from the iterative power-gradient approach to a full-range scan and peak-extraction method, allowing the optimum within the predefined scan range to be identified in a single pass without repeated iterations. Although a full-range scan requires scanning the predefined range for each axis, it reduces the need for repeated iterative searches and identifies the optimum within a single pass in all reported experiments. Accordingly, the reported alignment time decreased from 238.5 s with an average of 4.13 iterations in the previous system to approximately 59 s in the present system, despite the increase in controlled axes from four to seven. In addition, the measured power profiles exhibited a single dominant peak, consistent with the expected Gaussian-like overlap between the focused beam and the single-mode fiber, and competing local maxima were not observed within the tested scan ranges. Although a direct performance comparison is difficult due to different experimental configurations, the expanded alignment capability and improved optimization performance can be observed in the present system.

A key feature of the proposed algorithm is that peak relocation is performed based on optical-power feedback rather than stored actuator coordinates. This is important because open-loop piezo actuators can exhibit hysteresis and positioning errors when the same command is reapplied [51,52], and piezo motors may also be affected by friction-dependent variations [53,54]. Therefore, simply recording the coordinate at which the maximum power is observed and returning the actuator to that stored coordinate can lead to alignment error. In the proposed algorithm, however, the maximum optical power is first extracted from the scan profile, and the actuator is then moved back while the photodetector signal is monitored in real time. The actuator stops when the measured optical power reaches the extracted maximum power. In this way, the alignment is closed-loop with respect to the coupled optical power, even though the actuator position itself is controlled in an open-loop manner. This is particularly suitable for single-mode fiber coupling, because the coupled optical power directly reflects the alignment between the focused beam and the fiber core.

The linear relationship between delivered optical power and PA image quality observed in the phantom and human experiments further supports the use of optical power as the feedback variable for automated coupling. In the leaf-skeleton phantom, both the PA amplitude and SNR increased with the delivered optical power, confirming that the optical-power feedback measured during alignment is an appropriate control variable for optimizing image quality. In vivo human finger imaging showed a similar trend, with clearer visualization of superficial microvascular structures when the delivered power increased. These results indicate that the proposed module can improve coupling stability and image quality not only in controlled phantom experiments but also in biological tissue imaging, where scattering and motion-related factors may be present. The repeatability experiments further demonstrated that the proposed alignment sequence could reach a stable coupling condition from different initial misalignment conditions. The consistent final coupled power and repeated phantom images also support that the extracted peak positions corresponded to reproducible optimized coupling conditions within the tested scan range.

However, several limitations remain. The current implementation requires a coarse manual offset before core alignment because the piezo-driven stage cannot be automatically driven in the negative direction owing to hardware limitations. This limitation could be addressed by using a bidirectional controller or an actuator with a wider travel range. Although the system successfully established coupling from a nearly decoupled condition with an initial optical power of 0.67 nW, this value should not be interpreted as the minimum recoverable power of the system. Systematic evaluation of lower initial coupling conditions was experimentally difficult. A more sensitive PD or a wider scan range may further improve recovery from more severely misaligned states. Slight decreases in coupled power were occasionally observed after axes aligned later in the sequence, likely because pulse-to-pulse laser fluctuation could cause a transiently elevated reading to be selected as the peak. Because the second mirror contributed only marginally after core alignment and first-mirror steering, shortening the selected mirror scans may reduce the total alignment time in future implementations. Future studies will further evaluate the system under controlled vibration and long-term thermal-drift conditions to quantify its ability to maintain or recover coupling under practical environmental disturbances. The present optimization strategy was specifically developed for OR-PAM systems using single-mode fiber delivery. In multimode fibers, excitation of multiple guided modes may produce more complex coupling profiles with multiple local maxima. Therefore, the current peak-detection strategy may require modification for reliable optimization in multimode fiber-based systems.

Nevertheless, the proposed approach may be useful beyond OR-PAM, because free-space-to-fiber coupling is widely used in optical biomedical imaging and spectroscopy systems. By integrating mirror-based beam steering with three-axis fiber-core alignment, the proposed module provides a practical strategy for automated coupling without requiring position sensors. The demonstrated improvements in coupled optical power and PA image quality suggest that this approach can enhance the transportability, robustness, and usability of fiber-coupled OR-PAM platforms and other optical imaging systems.

## Data Availability

The data presented in this study are available on request to the corresponding author.

## Supplementary Materials

The following supporting information can be downloaded at: https://www.mdpi.com/article/10.3390/s26206469/s1, Figure S1: The measured optical power during automated laser-fiber coupling optimization with different alignment sequences. Figure S2: Measured optical power during automated laser-fiber coupling followed by repeated core alignment. Figure S3: Photograph of the transportable OR-PAM system used for the relocation experiment. Table S1: Detailed part list and key specifications of the automated laser-fiber coupling module integrated with the OR-PAM system. Table S2: Comparison of the alignment configuration and optimization performance between the previously reported mirror-based laser-fiber coupling system and the present system. Algorithm S1: Laser-fiber coupling optimization.
