# Supplementary material for: Automated Laser-Fiber Coupling with Three-Axis Fiber-Core Alignment for Optical-Resolution Photoacoustic Microscopy
Source: Sensors (Basel). 2026 Oct 13;26(20):6469. doi: 10.3390/s26206469 (PMC13709162; doi:10.3390/s26206469)
Supplement: Supplementary file 1 [file sensors-26-06469-s001.zip › sensors-4594360-supplementary.pdf]

Supplementary Information for

Automated Laser-Fiber Coupling with Three-Axis Fiber-Core Alignment for Optical-Resolution Photoacoustic Microscopy

Yongjae Jeong[1], Dongbin Lee[1], Haeni Lee[1], Hwidon Lee[1,2], Jinwoo Yoo[3], and Jeesu Kim[1,*]

[1] DDepartment of Cogno-Mechatronics Engineering, School of Transdisciplinary Engineering, Pusan National University, Busan 46421, Republic of Korea; zxc2726655@pusan.ac.kr (Y.J.); ehdqls3367@pusan.ac.kr (D.L.); haenilee@pusan.ac.kr (H.L.); hwidonlee@pusan.ac.kr (H.L.)

[2] Engineering Research Center for Color-Modulated Extra-Sensory Perception Technology, Pusan National University, Busan 46241, Republic of Korea

[3] Department of Automobile and IT Convergence, Kookmin University, Seoul 02707, Republic of Korea; jwyoo@kookmin.ac.kr

***Corresponding author:** jeesukim@pusan.ac.kr

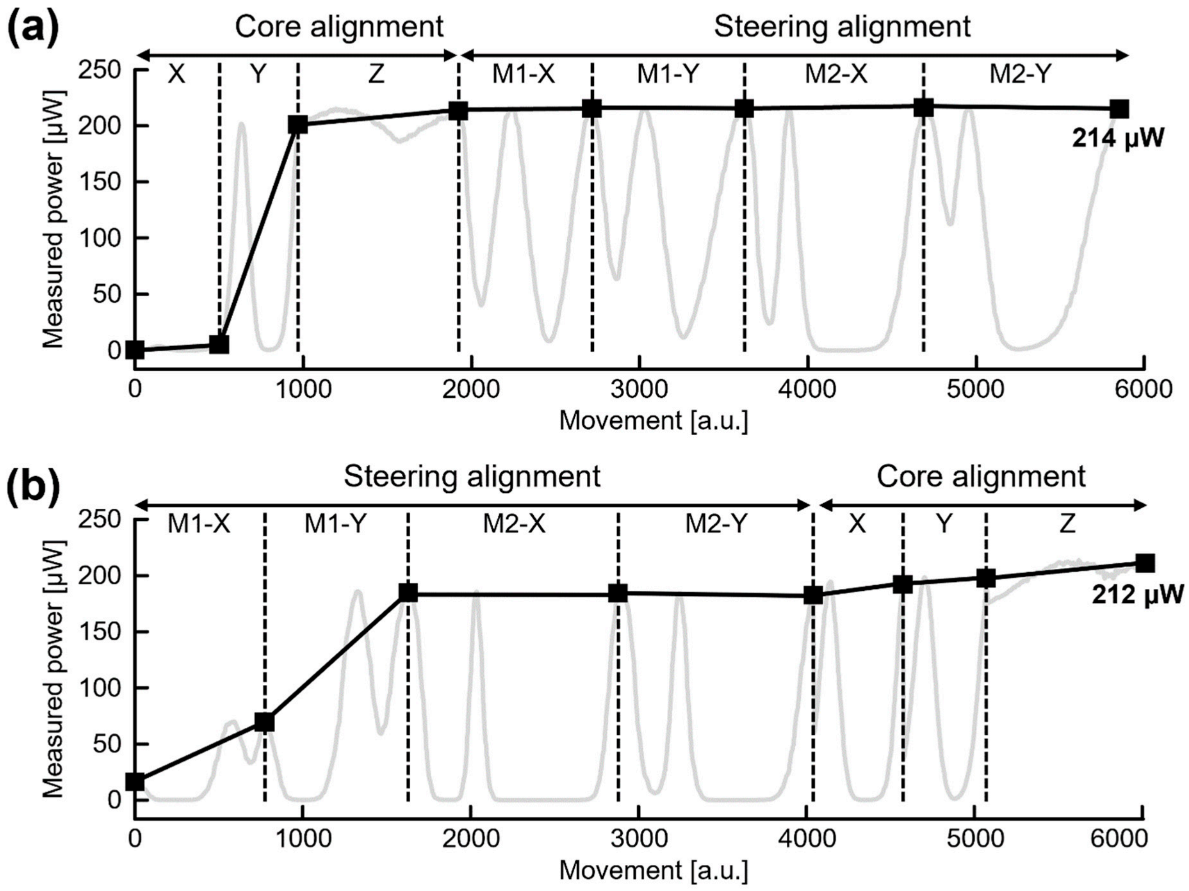

Figure S1. The measured optical power during automated laser-fiber coupling optimization with different alignment sequences. (a) Core alignment followed by steering alignment. (b) Steering alignment followed by core alignment. Despite the different alignment orders, comparable final coupled optical powers were obtained. The experiments were performed under the same laser output power.

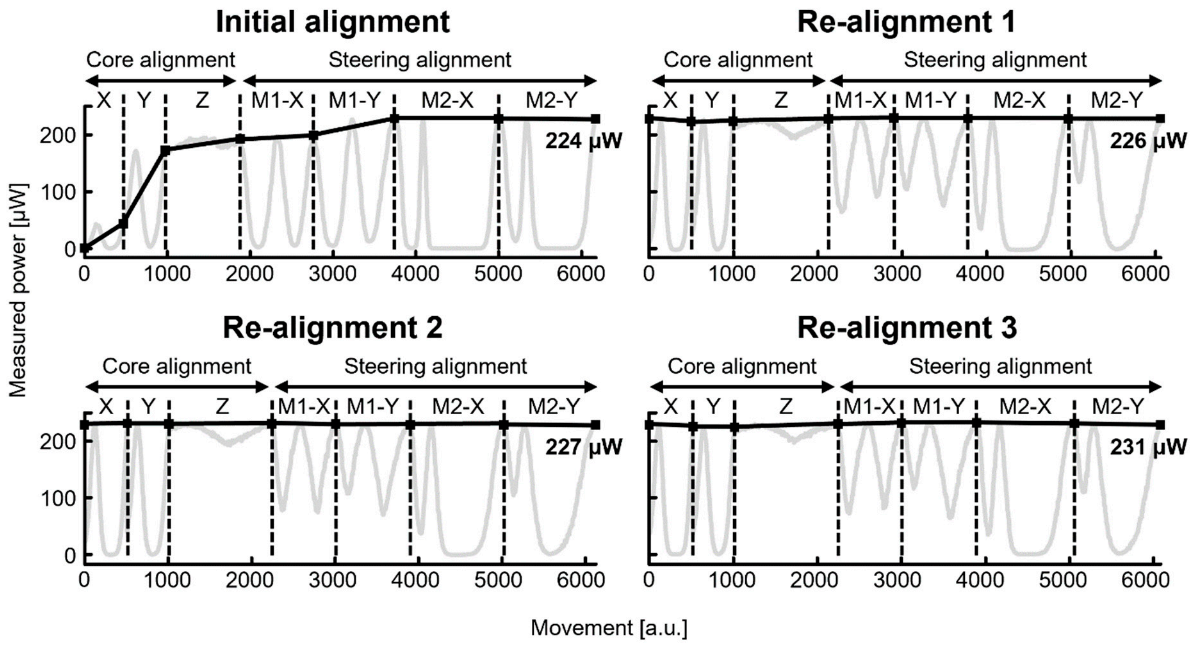

Figure S2. Measured optical power during automated laser-fiber coupling followed by repeated core alignment. After completion of the initial seven-axis alignment, the core-alignment sequence was repeated. The final coupled optical power remained unchanged (227 ± 2.94 μW), supporting the conclusion that the initial alignment had already reached an optimal coupling condition.

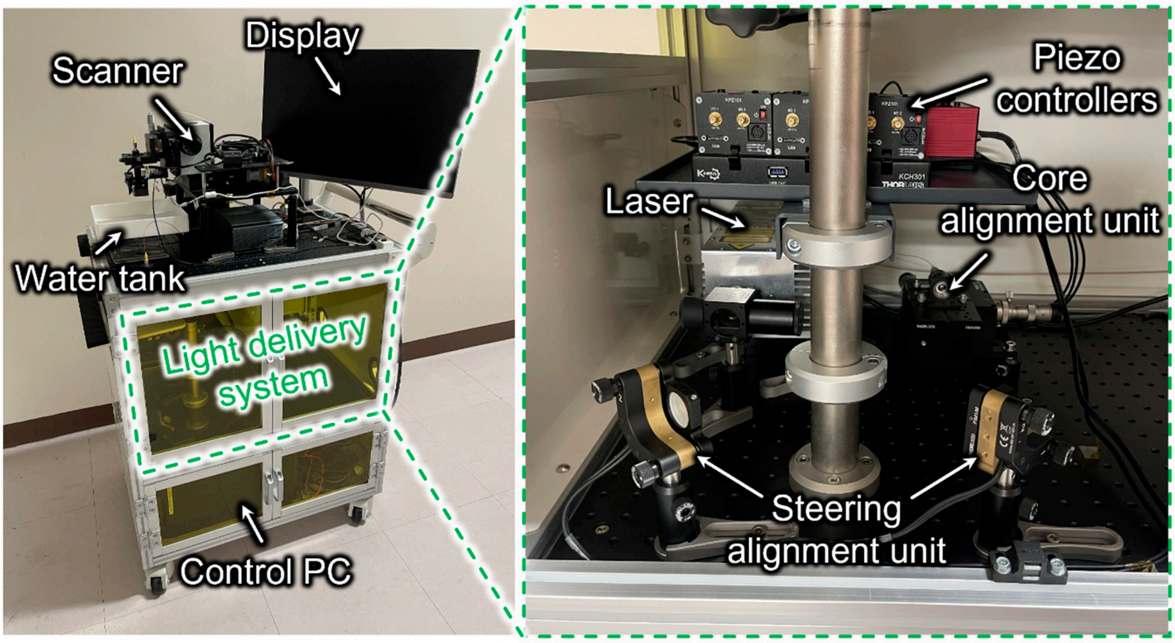

Figure S3. Photograph of the transportable OR-PAM system used for the relocation experiment. The system includes the scanner, water tank, control PC, and light-delivery section. The enlarged view shows the automated laser-fiber coupling module integrated in the light-delivery section. OR-PAM, optical-resolution photoacoustic microscopy; PC, personal computer.

Table S1. Detailed parts list and key specifications of the automated laser-fiber coupling module integrated with the OR-PAM system. OR-PAM, optical-resolution photoacoustic microscopy; DAQ, data acquisition; PRF, pulse repetition frequency; NA, numerical aperture; SMF, single-mode fiber.

Function	Component	Model	Manufacturer	Key specification
Light Delivery	Laser	DX-532-2	Photonics Industries, USA	Output wavelength: 532 nm<br>PRF: 20 kHz
	Mirror mount	PIM1	Thorlabs, USA	Angle range: ±2 deg
	3-axis stage	MAX301	Thorlabs, USA	Piezo travel range: 20 µm
	Mirror	PF10-03-G01	Thorlabs, USA	Reflectance: 92% @ 532 nm
	Objective lens	LMH-20X-532	Thorlabs, USA	Focal length: 10 mm<br>NA: 0.40
	Fiber collimator (coupling)	F220FC-532	Thorlabs, USA	Focal length: 7.86 mm<br>NA: 0.51
	Optical fiber	460HP	Thorlabs, USA	Single-mode operating range: 450–600 nm
Power Monitoring	Optical coupler	TN532R1F1	Thorlabs, USA	99:1 single-mode coupler
	Photodetector	S140C	Thorlabs, USA	Detection wavelength: 350–1100 nm
	Power meter	PM100D	Thorlabs, USA	Sampling rate: 3 kHz
Scanner	Fiber collimator (scanner)	F220FC-532	Thorlabs, USA	Focal length: 7.86 mm<br>NA: 0.51
	Objective lens	AC127-050-A	Thorlabs, USA	Focal length: 50 mm
	Ultrasound transducer	OptichoM-RUT	Opticho, Korea	Focal length: 18.6 mm<br>Center frequency: 25 MHz
	Prism	MRA10-G01	Thorlabs, USA	Reflectance: >90%
	Galvanometer mirror	GVS001	Thorlabs, USA	Scanning angle: ±12.5 deg
	Water tank	-	Custom	Dimensions: 250×100×50 mm^3
Control and Acquisition	OR-PAM	OptichoM	Opticho, Korea	Lateral resolution: ~10 µm
	Controller (mirror mount)	KIM101	Thorlabs, USA	Four-channel
	Controller (3-axis stage)	KPZ101	Thorlabs, USA	Output voltage: 0 – 75 V
	DAQ	ATS9352	AlazarTech, Canada	Sampling rate: 500 MHz<br>Precision: 12-bit

Table S2. Comparison of the alignment configuration and optimization performance between the previously reported mirror-based laser-fiber coupling system and the present system. PD, proportional-derivative control.

		**Previous System**§	**Present System**
**Number of Automated Axes**	**Total**	4	7
	**Beam Steering**	4<br>(2 angular axes × 2 mirrors)	4<br>(2 angular axes × 2 mirrors)
	**Core Alignment**	0	3 (x, y, and z axes)
**Alignment Approach**	**Automated Alignment**	Beam steering only	Beam stearing +<br>Core alignement
	**Initial Alignment**	Manual core alignment required	Manual coarse initialization
	**Optimization Strategy**	Iterative PD-based optimization	Full-range scan +<br>Peak extraction + Relocation
**Optimization Performance***	**Number of Iterations**	4.13 (average)	1 (single pass)
	**Optimization Time**	238.5 s (average)	~59 s
	**Final coupling Efficiency**	~22.5%	~39%

* Reported results obtained under different experimental configurations

§ Han et al., *Sensors*, 2023, 23(14): 6643.

Algorithm S1. Laser-fiber coupling optimization.

Initialize:
    $R[a]$ = scan range for each axis a
    $\delta[a]$ = step size for each axis a

For a = 1 to 7 **Do**
    Initialize the actuator position for the current axis
    ScanCount = 0;   ValidPeak = False;   PeakValue = 0

    While ScanCount < Predefined Maximum ScanCount **Do**
        For each position x in $R[a]$ with step size $\delta[a]$ **Do**
            Move current axis to x
            $P(x)$ = Average 15 photodetector signals
        End

        $\mathcal{P}(x)$ = Smoothed $P(x)$ with a 10-point moving average
        $\mathcal{P}'(x)$ = Derivative of $\mathcal{P}(x)$
        For each x **Do**
            If $\mathcal{P}'(x)$ changes from positive to negative
                ValidPeak = True
                PeakValue = $\mathcal{P}(x)$
            End
        End

        While Relocated axis to initial position **Do**
            P_r = Average 15 photodetector signals
            If P_r = PeakValue
                Finish relocating and break while loop
            End
        End

        If ValidPeak = True
            Mark axis a as optimized and break while loop
        Else
            Update $R[a]$ around the relocated position
            ScanCount = ScanCount + 1
        End
    End
End
